# Structure learning and the Occam's razor principle: a new view of human function acquisition

**DOI:** 10.3389/fncom.2014.00121

**Published:** 2014-09-30

**Authors:** Devika Narain, Jeroen B. J. Smeets, Pascal Mamassian, Eli Brenner, Robert J. van Beers

**Affiliations:** ^1^Max Planck Institute for Biological CyberneticsTuebingen, Germany; ^2^Max Planck Institute for Intelligent SystemsTuebingen, Germany; ^3^Faculty of Human Movement Sciences, MOVE Research Institute Amsterdam, VU UniversityAmsterdam, Netherlands; ^4^Laboratoire des Systèmes Perceptifs (CNRS UMR 8248), Ecole Normale SupérieureParis, France

**Keywords:** structure learning, function learning, Bayesian model selection, Occam's razor, sensorimotor learning

## Abstract

We often encounter pairs of variables in the world whose mutual relationship can be described by a function. After training, human responses closely correspond to these functional relationships. Here we study how humans predict unobserved segments of a function that they have been trained on and we compare how human predictions differ to those made by various function-learning models in the literature. Participants' performance was best predicted by the polynomial functions that generated the observations. Further, participants were able to explicitly report the correct generating function in most cases upon a post-experiment survey. This suggests that humans can abstract functions. To understand how they do so, we modeled human learning using an hierarchical Bayesian framework organized at two levels of abstraction: function learning and parameter learning, and used it to understand the time course of participants' learning as we surreptitiously changed the generating function over time. This Bayesian model selection framework allowed us to analyze the time course of function learning and parameter learning in relative isolation. We found that participants acquired new functions as they changed and even when parameter learning was not completely accurate, the probability that the correct function was learned remained high. Most importantly, we found that humans selected the simplest-fitting function with the highest probability and that they acquired simpler functions faster than more complex ones. Both aspects of this behavior, extent and rate of selection, present evidence that human function learning obeys the Occam's razor principle.

## Introduction

Identifying relationships among environmental variables is often crucial to accurately predicting the value of one variable while using information from another. For instance, we routinely judge whether to cross a road after a quick glance at an oncoming car because we make predictions consistent with the functional relationship between distance, velocity and time. While playing ball sports, we predict how to best intercept a moving ball aided by predictions based on limited visual information of its trajectory. When trained, humans can reproduce these functional relationships fairly accurately. An explanation of how humans learn to accurately reproduce functions is however widely contested.

Some key issues underlying the study of function learning are, abstraction, rule-based learning, and parsimony. Abstraction refers to the ability to observe low-level information and infer an overarching rule that helps to better classify and predict these observations. Rule-based learning, in the context of function learning, refers to whether humans make principled assumptions while interpolating or extrapolating functions. Parsimony refers to the preference of a learning method that is effective in producing reliable predictions yet requires minimal resources.

Some theories suggest that functions are abstracted as integrated entities in a manner similar to polynomial regression (Carroll, 1963; Brehmer, 1974; Koh and Meyer, 1991) while other theories assume that no abstraction is necessary to explain human function learning. The proponents of the latter view propose that localized learning of a function through multiple independent elements (Kalish et al., 2004), Gaussian Processes (Griffiths et al., 2008) or paired associations between input and output based on trial and error learning (Busemeyer et al., 1997; DeLosh et al., 1997; McDaniel et al., 2009) can explain responses produced by humans. The second issue of contention is whether function learning is rule-based (irrespective of abstraction of the function) or whether function learning is a consequence of simple associations that emerge through trial-and-error learning. For instance, some algorithms assume that local linear basis functions can partition any given function until it is reasonably approximated. The use of a linear basis function constitutes a rule-based assumption. Thirdly, various algorithms proposed in function learning use a different number of parameters and therefore differ in algorithmic complexity, while at the same time, the generating functions presented to participants also differ in complexity since they have a different number of parameters (see McDaniel et al., 2009). Under these circumstances, it becomes difficult to compare models in an unbiased manner and at the same time detect systematic differences that humans display toward functions with different complexity. Without resolving such issues, the study of online learning of changing functional relationships will remain a challenge and it will be nearly impossible to tease apart whether a given effect is a consequence of inherent bias in algorithms or is derived from human behavior during online function learning.

In this work, we propose to study function learning under a new framework that provides a unifying perspective on all three of the above mentioned issues: *structure learning* (Braun et al., 2009, 2010a; Kemp and Tenenbaum, 2009; Tenenbaum et al., 2011). Structure learning supports the view that (1) functions are abstracted while at the same time, (2) allowing room for both associative and rule-based accounts for learning. (3) Assuming a structure hierarchy between functions and parameters allows us to study function learning online by internally compensating for algorithmic complexity, and therefore revealing the human treatment of function complexity while adjusting to online changes in the presented function.

Structure learning has recently emerged as an important theory to explain human learning in cognitive science (Kemp and Tenenbaum, 2008, 2009; Griffiths et al., 2010; Tenenbaum et al., 2011), perceptual learning (Körding et al., 2007; Braun et al., 2010b; Turnham et al., 2011; Narain et al., 2013a), and sensorimotor learning (Braun et al., 2009, 2010b; Acuña and Schrater, 2010). Its main principle is abstraction along an hierarchy of variables. It contends that the rapidity of learning and the extensiveness of learning generalization in humans can be explained by the abstraction of lower-dimensional manifolds. For example, in the case of function learning, a set of limited discrete function hypotheses may exist on a low dimensional subspace (ex. linear, quadratic or cubic), whereas the parameter spaces of each of these lie at a higher dimension (2 dimensions for linear, 3 dimensions for the quadratic). One of the advantages of such structure learning is that if evidence for a certain function is substantial, such information will constrain the search of parameters from an infinite search space to a finite subset within these higher dimensions and therefore facilitate the discovery of the true parameters. Thus, far, function-learning research has never attempted to separate these two levels of abstraction. Therefore, when participants did not reproduce the exact function, it was concluded that they could not learn the function. Although function learning and parameter learning may depend on each other, it should be possible to abstract the correct function without a completely accurate understanding of the parameters of that function.

Here we develop a new intuitive paradigm for function learning that introduces uncertainty into the learning process, thereby making multiple function hypotheses possible. Participants were given a brief spatial cue and were asked to shoot at a transient target that would appear after an unknown time-interval. Unbeknownst to the participants, the cue location and target time were related according to various continuous functions. In our first experiment, we tested performance based on predictions of associative learning algorithms (ALM: Associative learning model and EXAM: Extrapolation-Association model Busemeyer et al., 1997; DeLosh et al., 1997), on partition-based algorithms [POLE: Population of linear experts; (Kalish et al., 2004)], polynomial regression (Carroll, 1963; Koh and Meyer, 1991), and Gaussian Processes (Griffiths et al., 2008). Most importantly, we did not merely test the mean of the predictions, but also took the variance of the predictions of each of these algorithms into account. This method allowed us to assess how much of the variability in participants' responses could be explained by each algorithm.

The results of this study revealed that participants may be abstracting the functions that we presented. Motivated by this finding, we assumed a hierarchy between functions and parameters and used Bayesian model selection (BMS) to separate the model-learning and parameter-learning levels of analysis. BMS is a method to compute relative posterior probabilities among models by comparing the likelihood that each model produced the observed data (Raftery, 1995; Wasserman, 2000; Burnham and Anderson, 2002). The most important attribute of BMS is that it requires each parameter to be integrated out from the likelihood of each model. This gives rise to the marginal likelihood (or evidence) for that model, which is the key term in determining the model posteriors. When multiple models can fit the data equally well, models with more parameters have broader marginal likelihoods than those of simpler models. Therefore, BMS has an inbuilt parsimony mechanism that penalizes model hypotheses that have more parameters. At the same time, we implement BMS without any free parameters and ensure that all model hypotheses are equiprobable *a priori*. This ensures that the probabilities generated by BMS are largely driven by the data of the participants. This enables us to focus on the results of participants' behavior and to determine whether human learning is governed by any perceptual rules of parsimony based on function complexity. We ensure that other aspects of our analysis do not bias our findings by performing a control analysis where we quantify what to expect from a simulated participant with full knowledge of the structure in the stimuli.

When we analyzed our participants' data using BMS, we found that even when other functions were viable candidates, and even if parameter learning was not completely accurate, the probability of the simplest function that could account for the data was the highest. Furthermore, the rate of acquisition of this simpler function was faster than that of more complex functions. Such parsimonious selection and facilitation is reminiscent of the theoretical principle called the Occam's razor. The principle states that when different models of varying complexity can account for the data equally well, the simplest one should be selected.

## Materials and methods

### Experiment 1

#### Design and procedure

Seven naïve paid participants gave written informed consent to perform a computerized experiment. This experiment was part of a program that has been approved by the ethical committee of the faculty of Human Movement Sciences, VU University, and adheres to the principles expressed in the Declaration of Helsinki. Before starting the experiment, all participants were shown an instruction video that familiarized them with the protocol and showed an example of a trial. They were invited to perform five trials on the setup (display: 597 × 336 mm, 83 Hz) on stimuli that were not used in the experiment.

**Figure 1:**
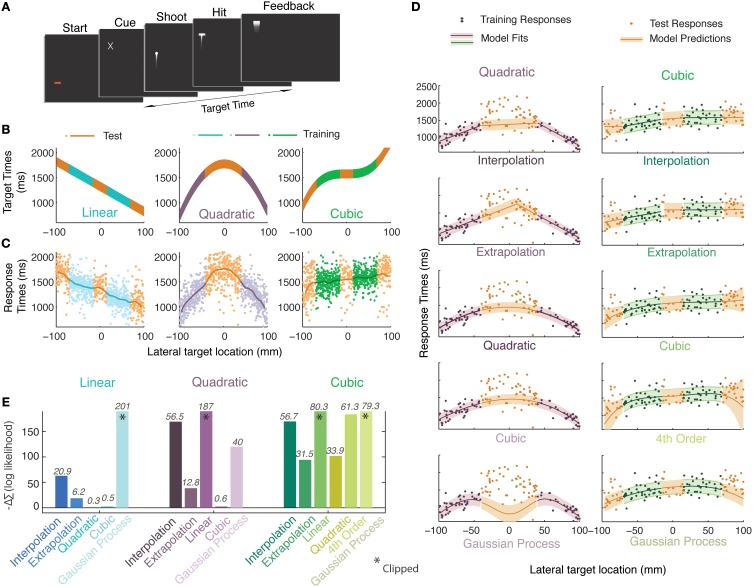
**Experiment 1**. **(A)** Timeline of a single trial. Participants initiated a trial, were presented with a cue about the spatial location of a future target and they pressed a key to launch an animated bullet to catch it en route. **(B)** Generating functions in three experimental sessions. The vertical extent of the curves indicates the time during which the target was visible (150 ms). Orange colors represent the test regions; other colors represent the training regions. **(C)** Smoothed data-averages (Gaussian kernel radius 10 mm) from all participants overlaid upon pooled responses. **(D)** A single participant's data for the quadratic (purple) and cubic (green) sessions along with the set of models/heuristics that were fit individually to the training data of each participant and whose prediction distributions were used to calculate likelihoods on the basis of the test data (curves indicate mean, shaded regions represent standard deviation). **(E)** Differences between the negative log likelihoods (summed over all participants) of the generating function and remaining heuristics and models. Positive bars indicate worse performance when compared to the generating function whereas negative bars indicate better performance. Numbers atop each bar quantify how well the model performs with respect to the generating function model i.e., 2lnK where K is the Bayes factor (see Materials and Methods for details).

In the study, participants attempted to strike a transient target by means of an animated bullet, launched on the screen by means of a key press (Figure 1A). Within a single trial, the animated bullet moved upwards and the target flashed at one fixed location. The lateral position of the target changed across trials. Before the start of each trial, participants saw a starting rectangle indicating the lateral coordinate of the future target, and pressed a key to initiate the trial. Upon initiation, the screen refreshed to a plain black background whereupon a cue flashed briefly (35 ms) at the exact location of the future target, serving as a second spatial cue of the lateral target position. Participants had to anticipate the target by firing an animated shot that would intercept the target, which flashed for 150 ms after an unknown interval. If the shot was fired after the target appeared it would surely miss (300 ms travel time on each trial).

Unbeknownst to the participants, there was a functional relationship between the time at which the target appeared after the start of the trial and the lateral position of the cue (identical to the lateral position of the target). In order to distinguish the presented functions from functions inferred from participant responses, we shall refer to the former as ‘generating functions’. Three such generating functions were presented in a different session each: a linear, a quadratic, and a cubic function spanning 200 mm of space and a temporal range of 660–2000 ms. Within a session the lateral positions of the stimuli were serially uncorrelated and uniformly distributed. In the equations, x represents the lateral position of the stimulus in mm, where *x* = 0 represents the center of the display and of all distributions. f(x) represents various functions specifying the onset time of the stimuli (in ms) relative to trial initiation:

$$\begin{array}{l} \text{Linear}:\;f_{1}(x)=-5x+1250\\ \text{Quadratic}:\;f_{2}(x)=-0.1x^{2}+0.5x+1700\\ \text{Cubic }(\text{wherea}=1/1500):\;f_{3}(x)=ax^{3}+ax^{2}-3ax+1500 \end{array}$$

There were two types of trials, those in which the target was shown (training trials) and those in which the target was never shown (test trials). In the training trials, the full extent of the error was observable since the bullet crossed the screen before the trial ended. Participants were given points, auditory and visual signals if they scored a hit on the training trials. The bullet was seen on test trials, but the target never appeared, and neither did any feedback. Training and test trials were drawn from separate regions (Figure 1B). Three sessions, separated by 5-min pauses, were each dedicated to a certain generating function. Different pre-trial screen colors were used to aid participants in their recollection of these sessions when they were questioned later. Participants were asked to treat each block as a new experiment. The session order was randomized across participants. Each session consisted of 170 trials in total, the first 50 were training trials and thereafter 60 test and training trials were interleaved.

At the conclusion of the experiment, participants were asked if they used a certain “strategy” to maximize their points. If they indicated that they recognized a relationship between the target location and timing, they were presented with a sheet containing 16 graphs (Supplementary Information Figure SI-1), consisting of functions of various shapes and forms, and asked to pick the generating functions in the correct order in the three sessions (also distinguished by different colors) in which they were presented.

#### Data analysis

Of the seven participants that we tested, we excluded from the analysis the data of one participant who secured an average hit rate of less than 10% for all three sessions and responded at roughly the same value for the duration of each session, thereby demonstrating no knowledge of the nature of the generating function. The lowest average hit rate among the remaining six participants was 24%, while the mean hit rate was about 38%.

To the training responses of the remaining six participants, we fitted several models and heuristics that were inspired by various function-learning accounts in the literature. The parameters of all of the following models were fit in the same way only on the training region data, through maximum likelihood estimation. (1) An *interpolation* heuristic was fitted (details of fitting follow in next paragraph) with separate line segments for each training region and the predictions were interpolated linearly between these. In case of extrapolation regions (linear and cubic functions), this algorithm produced flat flanks extending at a constant value obtained from the nearest peripheral observations of the training range. This heuristic was developed to account for the behavior of algorithms like the associative learning model (ALM: Busemeyer et al., 1997) and for findings in studies where participants used linear approximations for interpolation but were not successful in extrapolating (Ernst and van Dam, 2010). We also used (2) an *extrapolation* heuristic that also fitted line segments to each training region but extrapolated its predictions. In the flanked test regions (central), when the intersection point of the two fitted line segments lay within the test region, we extrapolated these segments and when it did not, we linearly interpolated between the line segments. This is a partitioning algorithm based on Population of Linear Experts (POLE: Kalish et al., 2004). The associative ALM model was further developed to include linear extrapolation EXAM (DeLosh et al., 1997), and therefore uses different mechanisms to explain interpolation and extrapolation behavior. EXAM's predictions are therefore covered by a combination of the interpolation and extrapolation heuristic (discussed later).

To account for the function abstraction view either through polynomial regression or log-polynomial regression (Carroll, 1963; Brehmer, 1974; Koh and Meyer, 1991), we also used the (3) *generating function* and (4) *various polynomials of lower and higher degrees*. And finally, we used (5) Gaussian processes regression with a squared exponential kernel function (free parameters: scale and precision) to test a novel approach to function learning (Griffiths et al., 2008). We could have used different basis functions for each generating function like Griffiths et al. (2008), however, the use of various polynomial basis functions implies the abstraction of different functions and therefore would be tantamount to using the Structure learning framework. Therefore, we constrain our Gaussian processes regression analysis in a non-parametric and abstraction-free spirit using the most widely-used Gaussian processes kernel, the squared-exponential kernel.

We assumed, based on previous experiments (Narain et al., 2013a) that participants would have adequately learned the task in the first fifty training trials. We therefore removed these from the analysis. The remaining 60 training trials, which were interleaved with the same number of test trials, were used to fit the parameters for each function using maximum likelihood estimation. Using the fitted values of the parameters from the training regions, the mean and variance of predictive distributions were generated for the stimuli presented in test regions. The predictive distributions for continuous functions were assumed to be Gaussian and their mean and variance were calculated by calculating the variance of the model given parameters. For example, in the linear case, where ${\hat{\theta }}_{0}$, is the estimate of the constant (bias) parameter, and ${\hat{\theta }}_{1}$ that of the slope, while x^*^ represents the observed location of the cue for which a timing will be predicted, then the variance of that prediction is given by:

$$var\left({\hat{\theta }}_{0}+{\hat{\theta }}_{1}x^{*}\right)=var\left({\hat{\theta }}_{0}\right)+var\left({\hat{\theta }}_{1}\right)x^{{*}^{2}}+2x^{*}cov\left({\hat{\theta }}_{0},{\hat{\theta }}_{1}\right)$$

In the case of heuristics, different variances in two segments gave rise to discontinuities in the predicted variance. The Gaussian Processes provide their own predictive distribution. Given these predictive distributions, we then obtained the likelihoods of these heuristics and models given the responses in the test regions.

#### Bayes factor

The Bayes Factor (K) was calculated using the marginal likelihoods by integrating the likelihoods over a large range of parameters values for each model and heuristic. We then took the ratio of the marginal likelihoods of each model and heuristic against that of the generating function. The values reported in Figure 1E are 2 ln(K), which can be interpreted as in Table 1 (Kass and Raftery, 1995).

**Table 1:** **A scale to interpret the measure 2 ln(K), where K is the Bayes Factor, a measure used in Figure 1E**.

***2 ln(K)***	**Strength of evidence**
0–2	Not worth a mention
2–6	Positive
6–10	Strong
>10	Very strong

*Unlike hypothesis testing, there is no single criterion but graded levels of evidence to support one of two hypotheses*.

While Experiment 1 was designed to test whether human participants can learn the structure of an abstract spatio-temporal relationship at all, it tells us little about how fast they can learn this relationship, how resilient they are while switching to a new relationship, and whether they acquire different models at different rates. We addressed these issues in a second experiment, and describe methods for Experiment 2 below.

### Experiment 2

#### Design and procedure

Thirty-one naïve paid participants provided written informed consent to perform the same task as Experiment 1. The data from three participants who matched the poor hit rate criteria in Experiment 1 were excluded from the analysis. Unlike Experiment 1, feedback of errors was given on each trial and the full domain of the generating function was uniformly and independently sampled in the presented range. The generating functions used were constant, linear and quadratic spanning the same ranges as in Experiment 1 (in the equations below, x represents lateral stimulus position in mm, functions f(x) represent the stimulus onset-timing relative to trial initiation, in ms).

$$\begin{array}{l} \text{Constant}:\;f_{0}(x)=1350\\ \text{Linear }f_{1}(x)=7x+1350\\ \text{Quadratic }f_{2}(x)=-0.15x^{2}+0.1x+2100 \end{array}$$

**Figure 2:**
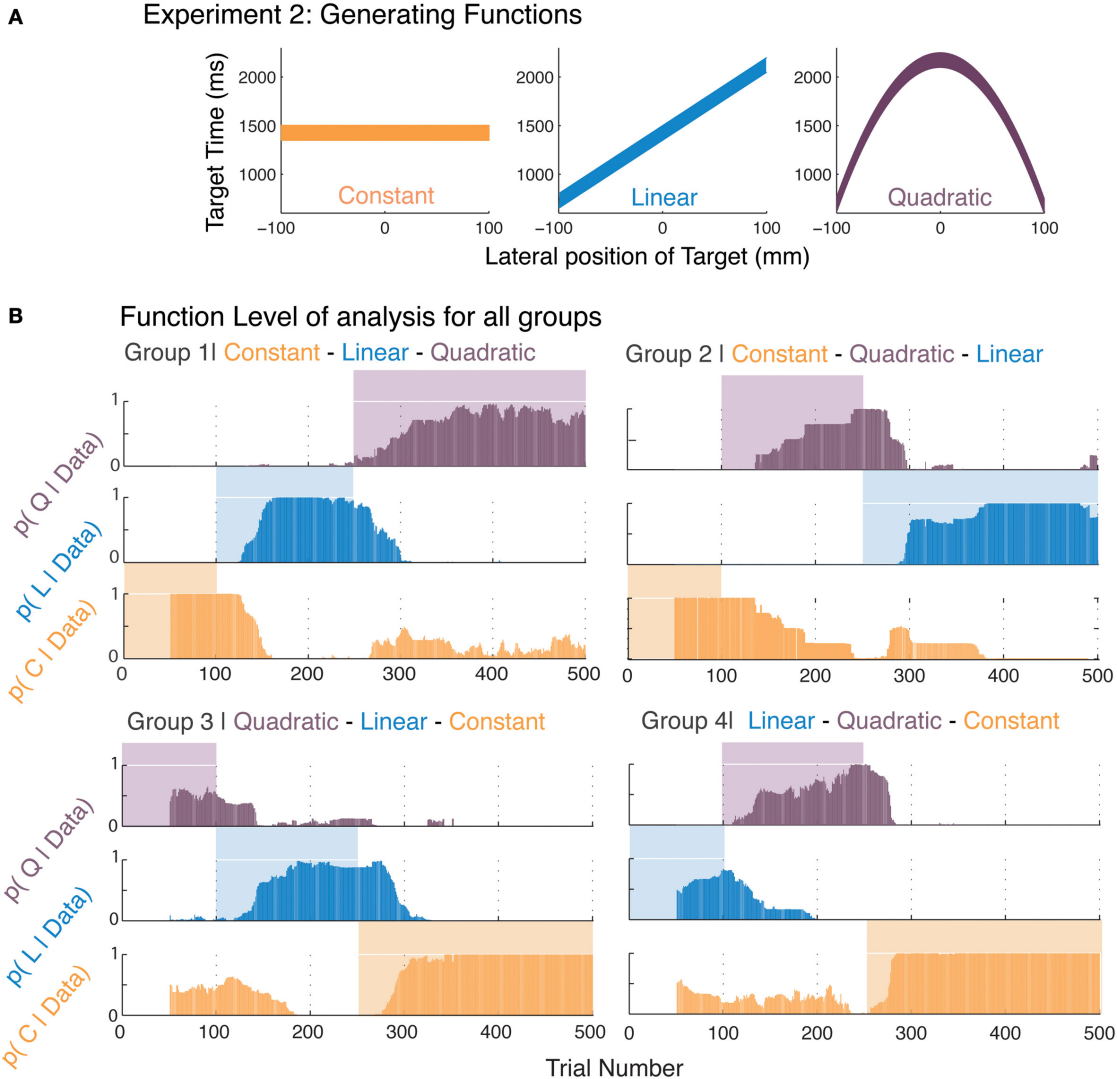
**Function learning in Experiment 2**. **(A)** The three generating functions used in this experiment were presented in different serial orders of presentation to the four groups. **(B)** The average posterior probabilities (foreground bars) for the most-likely function corresponding to participants' responses within a moving-window of 50 trials are shown over the course of the experiment. The generating function that was used at a certain trial is indicated by the presence of a background color. The four panels represent averages of the four groups with different presentation orders for the generating functions.

**Figure 3:**
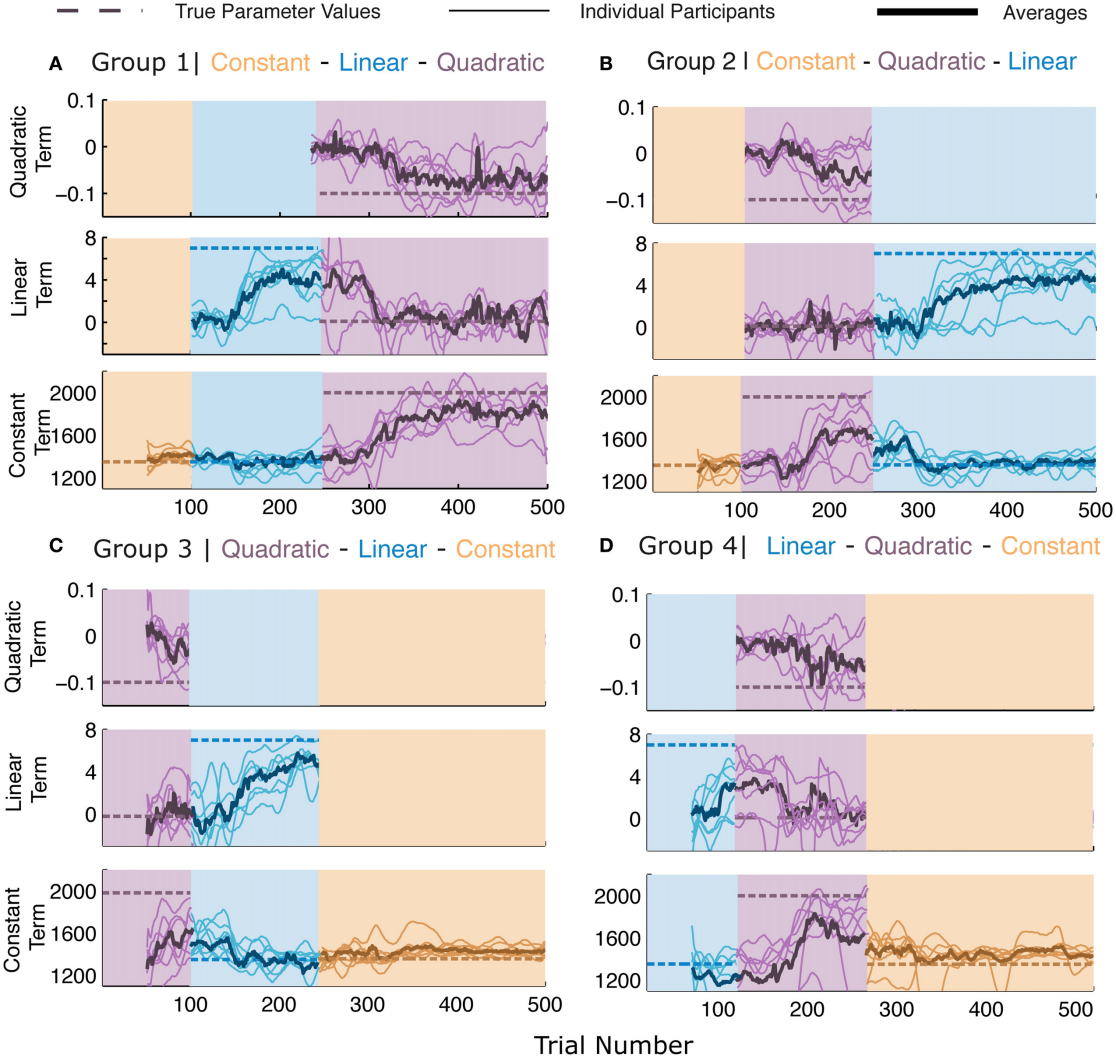
**Parameter learning in Experiment 2**. **(A–D)** The four panels represent the four different groups of participants. Each panel consists of three sub-parts, each indicating the parameters for the terms of the model when the corresponding generating functions were presented (note that the parameters have different scales and dimensions). Dashed lines represent the values of the generating function. All other lines represent the maximum likelihood estimate (MLE) of the parameters in a moving window of 50 trials. Thin lines represent individual participants and thick lines represent averages. The lines pertain to the parameters of the presented generating function and therefore there may be discontinuities in the lines at the switches. All participants' data are plotted irrespective of whether or not the generating function was the best description of the participants' responses.

During the course of the experiment, the generating function was switched twice, once after 100 trials and then after 250 trials. Participants were divided into four groups (after exclusions, there were seven participants in the first two groups, eight participants in the third group and six in the last group) with various kinds of switches (Figures 2, 3).

#### Data analysis and simulation

We analyzed both the model-level and parameter-level of analysis in moving windows of 50 trials (451 window frames for the 500 trials in total). This window-size was chosen based on a trade-off between the reduced power in smaller window sizes and loss of temporal resolution in larger ones. Rates of acquisition were calculated for 0.33, 0.5, 0.66, and 0.99 threshold probabilities of selection based on the posterior probabilities obtained from the *Model level of analysis*.

#### Control analysis

**Figure 4:**
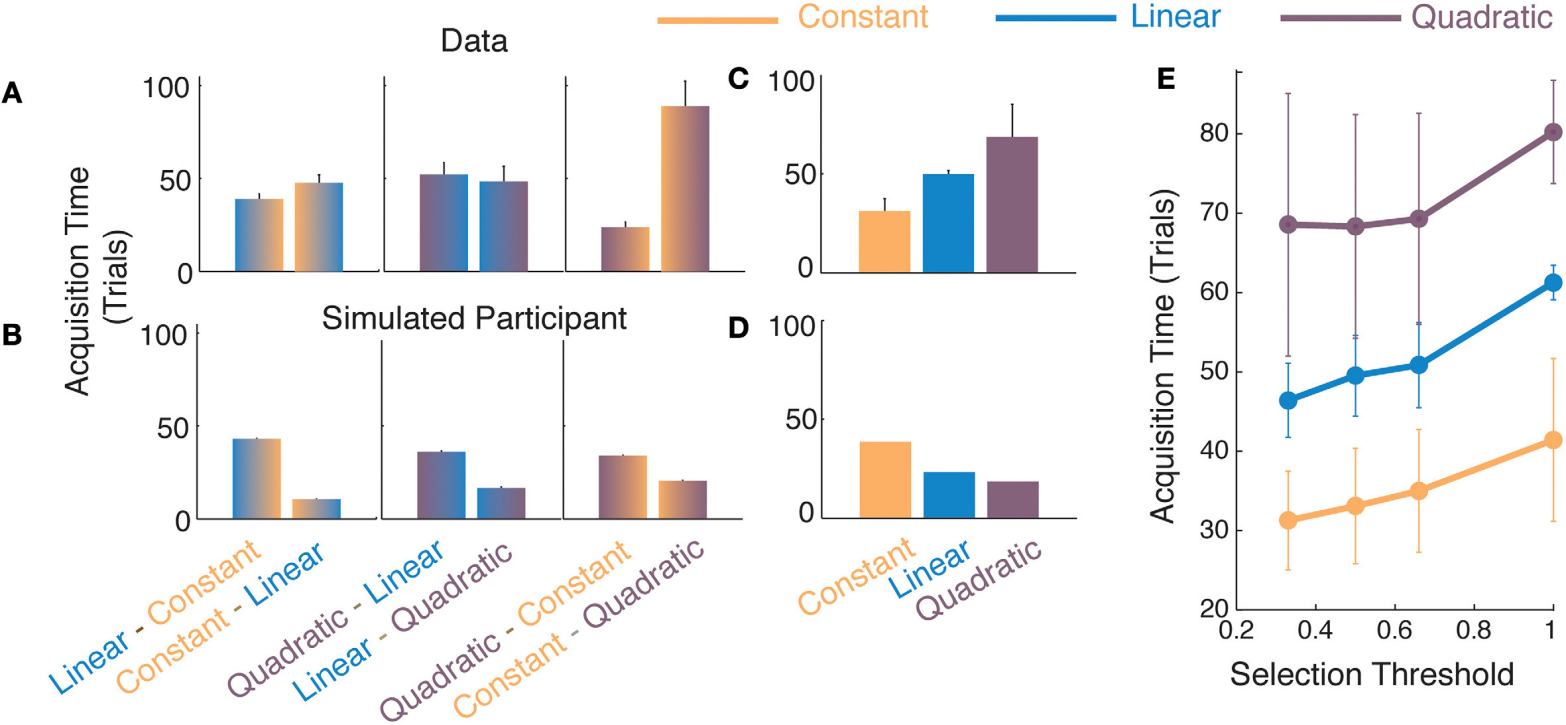
**Comparisons of function acquisition times in Experiment 2**. Comparison of **(A)** participants' data and **(B)** average responses from simulated participants with noisy responses (see Materials and Methods for various noise values) for different switches of generating functions. The ordinate represents acquisition time, i.e., the number of trials taken to achieve a selection probability of 0.33. Average acquisition times of each model for **(C)** participant's data and **(D)** for responses from a simulated participant. **(E)** Average function acquisition times for participants' data as a function of the selection threshold at which the acquisition time is determined. Error bars represent standard error across participants.

To control for whether the pattern of results was an inadvertent consequence of our analysis, we simulated the responses of a participant with noisy responses but perfect knowledge of the switches between models, the generating functions, and their corresponding parameters. Noise in responses plays an important role in such analyses and therefore we added different levels of zero-mean white Gaussian noise (sd: 10, 30, 50, 100, 150, 200, 400 ms) using Monte Carlo methods to the simulated participant's responses. We then performed a moving-window analysis identical to that used for the actual participants' data, in order to obtain the posterior probability of each model in each window frame. We found that even with different noise levels, the posteriors and acquisition times were almost identical as long as the maximum likelihood estimate (not the actual noise level) for the noise level was recalculated in every window (451 frames for 500 trials) for each run (total Monte Carlo runs = 500). In Figure 4, we use a simulated dataset with standard deviation 10 ms, which was the uncertainty caused by the refresh rate of the monitor.

#### Bayesian model selection

We used the Bayesian Model Selection framework to obtain the posterior probabilities of each model within a window frame. We computed the marginal distributions for each model by analytically integrating out any parameters from lower levels in the hierarchy. By using Bayes' rule, and assuming prior distributions (described below), we obtained posterior distributions for the models given the data.

The data that we obtained from participants were first cast as a multivariate Gaussian likelihood distribution (Equation 1). Here, Λ constituted a diagonal matrix where the precision (1/variance) of each element was specified by *l, observation noise*. Since we did not always have access to the value of *l*, we numerically marginalized this parameter (range of 0–5000 ms), assuming a uniform prior over this entire range. Therefore, our implementation of the BMS algorithm had no free parameters.

(1) The Likelihood of the models and parameters given the data for Experiment 2:
  $$\begin{array}{l} p(Data|\theta_{1:m},M_{j})=\frac{|\Lambda {|}^{1/2}}{{\left(2\pi \right)}^{n/2}}e^{-\frac{1}{2}{\left(Y-F(\theta_{1:m},X)\right)}^{T}\Lambda (Y-F(\theta_{1:m},X))}\\ \begin{array}{ll} n & \text{Number of observations}\\ m & \text{Numberof parameters in }j^{\text{th}}\text{ model}\\ M_{j} & j^{\mathrm{th}}\text{ model},\text{where }j\text{ = 1}-\text{3 models under}\\ & \text{consideration: constant, linear and quadratic} \end{array} \end{array}$$
  $$\begin{array}{ll} X\in {\mathbb{R}}^{n} & \text{Stimuli (lateral position)}\\ Y\in {\mathbb{R}}^{n} & \text{Observation vector (responses)}\\ F & \text{Generating function}\\ \Lambda & \text{Precision matrix for multivariate Gaussian}\\ & \text{Likelihood}\\ \theta_{i} & \text{Parameter space for each model} \end{array}$$

We defined priors over each of the parameters of the three generating functions (Equation 2). For simplicity, the Gaussian priors for the parameters were assumed to be independent of each other and centered at a mean value far from the true parameter value with their standard deviation scaled to an order of magnitude larger than their means. Thus, BMS did not have correct *a priori* information about the true mean of the parameters. It is important to note that the role of the prior in BMS is negligible in comparison to that of the marginal likelihood under these circumstances i.e., the mean value of the prior, due to its inflated variance hardly affects the outcome.

(2) Prior over parameters for Experiment 2:
  $$\begin{array}{l} p(\theta_{i}|M_{j})=\prod_{i=1}^{m}\frac{\sqrt{\omega_{i}}}{{\left(2\pi \right)}^{1/2}}e^{-\frac{1}{2}{\left(\theta_{i}-\mu_{i}\right)}^{2}\omega_{i}}\\ \begin{array}{ll} \omega_{i} & \text{Precision of the }i^{\text{th}}\text{ parameter distribution }(\text{1/variance})\\ & \text{of the }j^{\text{th}}\text{ model} \end{array}\\ \begin{array}{cc} \mu_{i} & \text{Mean of }i^{\text{th}}\text{ parameter distribution } \end{array}\\ \begin{array}{cc} \theta_{i} & i^{\text{th}}\text{ parameter vector of }j^{\text{th}}\text{ model where }j\text{=1}-\text{3} \end{array}\\ \begin{array}{cc} & \text{models under consideration: constant, linear and} \end{array}\\ \begin{array}{cc} & \text{quadratic} \end{array} \end{array}$$

For the posterior probability of models described in Equation (3), we first calculated the marginal likelihood by integrating the product of the likelihood from Equation (1) with the parameter priors from Equation (2).

(3) Marginal Likelihood for Experiment 2:
  $$\begin{array}{l} p(Data|Model)=\int_{\theta_{m}}\ldots \int_{\theta_{1}}p(Data|\theta ,Model)\\ \;p(\theta |Model).d\theta_{1}\ldots d\theta_{m} \end{array}$$

After each parameter was integrated out analytically, we used the marginal likelihood to compute the posterior probability of each model given the data (Equation 4). Here the prior probability of each model being selected was assumed to be uniform (equi-probable), therefore no one model was more likely than the other *a priori*.

(4) Posterior Distribution of models in Experiment 2:
  $$p(Model|Data)=\frac{p(Data|Model)p(Model)}{\sum_{i=1}^{3}p(Data|Model_{i})p(Model_{i})}$$

#### Parameter estimation

In addition to analyzing the model posteriors in Experiment 2, we also analyzed the learning of the parameters over the course of the experiment. We obtained maximum likelihood estimates of each parameter in each model given the observation noise that maximized the marginals in each window frame, obtained through Bayesian model selection. The search space for parameters used to determine the likelihood distributions (two orders of magnitude larger than actual parameter dimensions) for terms of various degrees were as follows, constant term: −10,000:10,000 ms, linear term: −100:100 ms.mm^−1^ quadratic term: −10:10 ms.mm^−2^. The maximum likelihood estimates were calculated at each frame of a window of 50 trials, and thus computed over the course of the experiment. These curves were smoothed using a univariate Gaussian kernel with a radius of 5 data points.

## Results

### Experiment I

After being shown a brief spatial cue, participants were asked to respond by firing an animated bullet, aiming to hit a transient target en route. Unbeknownst to them, the location of the cue and the timing of the target constituted a functional relationship (linear, quadratic, or cubic; Figure 1B) and the purpose of the experiment was to train them on certain regions of this function and test transfer to other regions. The participants' responses (lines in Figure 1C) seem to approximate most of the presented functions. In Figure 1D we see an example of some models and heuristics of the quadratic and cubic sessions being fit to the responses from the training regions obtained from a representative participant. To assess model performance, the likelihoods of the various models were calculated given the *test* stimuli and participants' test responses, based on MLE estimates of parameters from the training regions. The best model or heuristic would maximize the likelihood of the predictive distributions when given the participants' test responses i.e., it would minimize the negative log likelihood. None of the models or heuristics enjoy an advantage due to having more parameters since they were fitted on regions that were different than those they were tested on. This procedure uses a principle similar to cross-validation techniques. The minimum negative log likelihood (summed over trials and participants) that was obtained in all three cases was that of the generating function, indicating that it was the best model in all cases. But this does not necessarily imply that it was an unequivocal winner. To understand the relative performances of the models, we subtract the summed negative log likelihood of the generating function model from that of each other model (Figure 1E).

Another measure that we use to quantify these differences is the Bayes factor (reported atop bars in Figure 1E). We calculate the ratio of evidence (Marginal likelihood) between the generating function and each other model or heuristic to obtain the Bayes factor (details in Materials and Methods). We report twice the log of the Bayes factor for each heuristic and model in Figure 1E where any value greater than 2 indicates positive evidence for rejecting that hypothesis in favor of the generating function (Table 1, also see Kass and Raftery, 1995). We find that the differences between predictions from the *higher degree* polynomial regression and those from regressing with the generating function are negligible for the linear and quadratic sessions. For all three sessions, participants' performance is best predicted by regression with polynomial functions of degree equal to or higher than the generating function (Figure 1E). For all sessions, we find that the *extrapolation* and *interpolation* predictions, predictions from lower degree polynomials and predictions of Gaussian process regression do not perform as well as the generating function. Based on these results, one can conclude that our participants' performance is either consistent with the use of the veridical function or a higher degree polynomial suggesting that function abstraction may have taken place.

We had another source of information to determine whether participants had abstracted the generating function. After the experiment, participants were asked whether they were able to formulate a reliable strategy and if they indicated that there was a relationship between the lateral target position and its timing, they were asked to identify the nature of these relationships in the order in which they were presented them from a panel of 16 cartoons (Figure S1, Supplementary Information). All six participants identified the existence of a relationship in the stimuli and also identified the linear and quadratic relationship in the correct order. Four of these picked a positively sloped line for the cubic condition and two participants chose a constant line for their choice of generating function in the cubic session. Their selection of a positively sloped line for the cubic session also seems to correspond with what can be seen in the averaged responses of participants for the cubic session in Figure 1C. On the other hand, in Figure 1E, we find that a cubic model best predicts the responses of the participants in the cubic session. It must be noted, however, that the fitted cubic functions do not resemble the true generating function in the stimuli. The participants' selection of a straight line in the questionnaire after the experiment, may suggest that the information we provided about the cubic was too noisy for them to be certain of its true shape and a line best described the observations. Alternatively, they might have been using a model that we did not test. We speculate that participants' inability to reproduce the actual cubic function may be due to the very limited extent to which we exposed the cubic generating function in the training regions. This seems quite plausible since we know from other studies that observations of target times and positions can be noisy (van Beers et al., 1999; Ernst and Banks, 2002; Alais and Burr, 2004; Brenner and Smeets, 2007; Faisal et al., 2008; Maij et al., 2009; Jazayeri and Shadlen, 2010; Narain et al., 2013b).

### Experiment II

We found in Experiment 1 that polynomial regression, often with the generating function, best explains the participants' responses in most cases. Furthermore, upon post-experiment questioning, participants correctly identified the shapes of the linear and quadratic functions. This shows that the performance of our participants was most consistent with an abstracted function, especially in the linear and quadratic cases. The structure-learning framework when applied to this problem would constitute a hierarchy between the function and its parameters, whereby the abstraction of a function would constrain the learning of its parameters. To study whether function learning and parameter learning can coexist, we performed an experiment (Experiment 2) where we surreptitiously changed the function over the course of the experiment.

We analyzed the time course of function-learning and parameter-learning independently for our participants. We determined analytical solutions for the marginal likelihoods specific to our generating functions using the Bayesian Model Selection framework (Raftery, 1995; Wasserman, 2000; Burnham and Anderson, 2002). We thus obtained posterior probabilities of each function, which were independent of the values of their parameters. This allowed us to determine the time course of function acquisition. These posterior probabilities were directly inferred from the participants' responses and indicated the probability of functions that were most likely to be used by the participants in generating their responses. Therefore, we took these posterior probabilities to reflect the functions that participants abstracted. By computing these posteriors over a moving window, we were able to obtain an insight into how participants relearned functions after we administered a sudden switch. Figure 2B shows that participants were able to switch to the changes in the generating function. The background colors in Figure 2B indicate the generating function and we see the average posterior probabilities in the foreground. In order to show the variability of participants within each group, we calculated the mean posterior value for each participant's data for all three models while that model determined the stimulus (including the first 100 trials where learning may have been incomplete). We then averaged this mean value of the posterior for each model across participants in each group (Supplementary Information Figure SI-2). Note that these represent the extent of model selection and not the rate of acquisition shown in subsequent figures.

Figure 3 shows the results of the parameter-level of analysis, which in combination with Figure 2, provides us with the complete picture of our participants' learning behavior. While the parameter estimates are calculated for each function, it is worth noting that higher order functions can fully mimic simpler functions whereas the reverse is not true. In Figure 3, the dashed lines represent the true value of the parameters for the three models and the background colors display the generating functions that were presented over the course of the experiment. We see here that the averages across participants (thick lines) begin to converge toward the dashed lines after the switches occur, indicating that the parameters selected by the participants were close to the veridical parameter values. Please note that different parameter values differ in dimension and impact the shape of the function in different ways. For instance, small fluctuations in the quadratic term, lead to large changes in the convexity or concavity of the quadratic function. Most importantly, we see that the parameter values for participants do not always reach the veridical values, while the function selection probabilities remain high. Bayesian model selection can only incur high posterior probabilities for a certain function when the responses and noise-level indicate it to be a clear winner. Therefore, function abstraction is possible even when parameter learning is not fully accurate.

Furthermore, since Bayesian model selection calculates posterior probabilities for all models simultaneously and compensates inherently for their complexity, it allows us to perform Bayesian model comparison on all three functions simultaneously. We find that posteriors calculated for participant's responses correspond to high selection probabilities for the simplest model that is viable for the data (Figure 2B). For example, in selecting the linear model, the parameters of both the linear and quadratic models yield the same result. In fact, had there been serious non-linearities in participants' responses, the posteriors would have been highest for the quadratic function. We find in Figure 2B, that there is a remarkable coincidence in high selection probabilities for the function presented. This is independently confirmed by the parameter values in Figure 3. This selection of the simplest most viable model when other functions were equally plausible is consistent with the Occam's razor principle. One could argue however, that these results are symptomatic of the fact that Bayesian model selection has an internal penalty on its marginals that controls for complexity. To address this issue, in the following paragraphs we discuss an independent measure to test whether human learning of function obeys the Occam's razor.

Figure 2B suggests that the time taken by participants to learn different functions varies. As we reasoned above, if we can establish that the selection of simpler models that are viable is also facilitated over the selection of more complex models, this would be further support for the view that learning is consistent with the Occam's razor principle. If we observe the acquisition time for the quadratic function in Group 2 (Figure 2B), and compare it to the opposite transition, the switch to the constant function from the quadratic in Group 4, we find the latter to be more rapid. To better quantify these acquisition times, we organized the various pairs of switches on the basis of function complexity, i.e., constant to linear vs. linear to constant, and so forth, and calculated the average time for which the posterior probability for the correct model for each participant reaches a certain threshold (*p* = 0.33; chance level for model selection).

The results in Figure 4A show that participants acquired simpler models faster than complex models. To interpret these results and to ensure that the pattern observed in the data was not merely an artifact created by elements of the analysis or algorithm, we simulated responses generated by a simulated participant who had perfect knowledge of the location of switches and the nature of the models and parameters. We added various levels of noise to the simulated participant's responses (not shown in Figure 4, details in Materials and Methods) and then subjected these responses to the same windowed analysis as that used for our participants in Figure 2B. We considered the acquisition times obtained from the simulated participant as a baseline to interpret actual participants' data. In Figure 4B, we observe that the baseline is biased toward selecting a complex model more rapidly than selecting a simple one. This bias is introduced due to our use of the moving-window analysis. When the window over which we analyze our data moves over a transition-point between two models, it contains a mixture of responses from two different models. Of the two, the model with higher degrees of freedom is likely to distinguish itself more prominently than the simpler model and is therefore likely to be selected faster by the algorithm. For example, 15 independent samples from a quadratic are likely to provide strong evidence of non-linearity even when 15 remaining samples suggest a noisy linear trend. The moving-window analysis therefore invariably creates an asymmetry in the acquisition times such that more complex models are selected faster. Please note that the asymmetry found in the data of human participants was exactly the opposite in nature (Figure 4A).

For the participants, the acquisition of complex models was slower than that of simpler ones, which can be seen in the averaged plots in Figure 4C. Treating the model acquisition times from the simulated participant responses as a baseline criterion, we performed two-tailed *t*-tests (since the effect could be in either direction) with the differences obtained from the participants' data (Figures 4A,B). We found all differences to be significantly different from those obtained from the baseline [LC-CL *t*_(5)_ = −13.04, *p* < 0.01; QL-LQ *t*_(6)_ = −9.39, *p* < 0.001; QC-CQ *t*_(5)_ = −7.80, *p* < 0.001]. In all cases, the difference in acquisition times for the transitions to complex models were longer than those for the transitions to simper models. To determine whether the pattern of results from the participants' data depended upon a certain *selection probability*, i.e., the posterior probability at which we compute the acquisition time, we repeated the above analysis for different threshold criteria (Figure 4E). We found that the result that the acquisition for transitions to simpler functions is facilitated over transitions to complex ones, holds for different selection probabilities. Therefore, not only is the simplest most viable function selected to the greatest extent by the participants, its acquisition is also facilitated over that for more complex models. These results in combination are suggestive that function acquisition obeys the Occam's razor principle.

## Discussion

In this paper, we propose structure learning as a candidate framework to explain human function learning. We make our case by tackling three issues central to function learning. (1) Can humans abstract functions? (2) Are rule-based learning and associative learning mutually exclusive? (3) Do humans employ principles of parsimony, like the Occam's razor while learning various functions?

### Abstraction

Early accounts of function learning claim that humans are capable of abstracting continuous functions (Carroll, 1963; Bedford, 1989; Koh and Meyer, 1991). Recent accounts have shifted the focus from testing performance in humans to the trial-by-trial learning of the generating function presented (Kalish et al., 2007; McDaniel et al., 2009). In Experiment 1, we generate predictions that are compatible with five different function learning algorithms in the literature. When we tested both the participants' responses in the test regions we found that polynomial regression of the veridical model or higher orders, best explained the data. We assumed that all these algorithms consistently applied a single principle during interpolation and extrapolation of the function and therefore, we could not directly test an important function learning algorithm, EXAM, which assumes associational learning for interpolation but linear extrapolation. However, the predictions of EXAM would be consistent with the extrapolation heuristic for the linear and cubic functions, and for the quadratic function, they would be most consistent with the interpolation heuristic (see McDaniel et al., 2009). For our data, neither of these heuristics performed better than the generating functions.

Intuitively, one would expect human behavior to reflect some continuity of prediction across the entire domain of the functions that humans experience and predict in. For example, one could expect the variance of human predictions to gradually increase as the location of extrapolation moves further away from the last observed point, but one would not expect an abrupt jump in the prediction variance just outside the experienced range of stimuli. Such discontinuities sometime occur while implementing POLE and EXAM. To address this problem, Gaussian processes may prove a good alternative both (Griffiths et al., 2008). Gaussian process regression has elements of rule-based and instance-based learning, and embodies a unifying principle and suffers no discontinuities in its predictions. It is also compatible with neural network implementations and therefore enjoys a plausible description at a biological level. What we achieve through Bayesian model selection can be achieved by assuming different polynomial Gaussian process kernels, however, this would also require the explicit assumption that the function is abstracted, and therefore amounts to the same argument that is made by us through BMS. Gaussian processes with a generic kernel (squared exponent) could not however explain the performance of our participants convincingly, suggesting that there may be some value in assuming abstracted basis functions.

It has been thought that polynomial and heuristic regression models are indistinguishable since they make predictions that cannot be teased apart, however, with adequate statistical power, the use of likelihoods and quantifying these results using the Bayes factor, one can sensitively discriminate the predictions of these models. As is the case with all multiple model comparisons, there can be no certainty about whether participants were using a model that was not included in the hypothesis set. However, within the hypothesis set considered, which in our opinions spans all major algorithms in function-learning research, polynomial regression, especially with the veridical function, emerges as the best candidate. These results in combination with the results from the questionnaires after the experiments imply that either the veridical function, or a function of higher order was abstracted by the participants.

### Rule-based learning vs. associative learning

In the structure learning account, rule-based learning and associative learning can in fact coexist, just like we demonstrate in Experiment 2 that high posterior selection probabilities can occur even when the parameter values are not fully learned. One possibility to reconcile these two views is that associative learning may take place until a function is abstracted, and thereafter the function can constrain the search for parameters in a rule-based manner. This may indicate a new interpretation of results in previous function learning studies; the inability to recreate a presented function exactly does not imply the lack of an understanding of that function.

One interesting insight that has emerged from recent data is that the manner in which a function is exposed to participants may influence whether they abstract a rule or whether they merely learn an associative map. Fulvio et al. (2013) propose that sparse sampling leads to associative mapping whereas dense sampling may lead to the abstraction of a function. Narain et al. (2013a) found that participants could not learn a function when it was exposed to them locally, whereas they rapidly learned it when the sampling was uncorrelated and dense. Ernst and van Dam (2010) found a lack of linear extrapolation behavior in a shape association task, however they used sparse sampling. All these results suggest that density and serial correlation of sampling may play a role in whether learning is rule-based or associative.

### Occam's razor

It has long been reported in literature that there is a primacy of linear functions in human function learning (Carroll, 1963; Deane et al., 1972; Brehmer, 1974; Bedford, 1989). In other words, humans learn linear functions faster than non-monotonic non-linear functions. It has also been shown that across different blocks, more complex functions take longer to be learned (McDaniel and Busemeyer, 2005).

In Experiment 2, we study the acquisition of functions of different complexity as they change surreptitiously over time. This is the first time that a function learning study has been performed in a non-stationary environment. Our use of Bayesian model selection allows us to perform a parallel comparison among the three functions without any fear of biased outcomes due to overfitting with more complex functions. The results demonstrate that even when other models could explain the data equally well (i.e., for the constant case, a linear and quadratic model can mimic a constant model), The selection probabilities were highest for the simplest model (Figure 2). Please note that this result stands out especially because we implement BMS without any free parameters and use an equal *a priori* selection probability for each model.

The data in Experiment 2 was analyzed over a sliding window of 50 trials. This window-size was selected because it traded-off reliable posterior calculations with the minimum loss of temporal resolution. This does not take the possibility that participants may have switched models multiple times during the transition, into account. Unfortunately, at smaller window sizes estimating the posterior becomes unreliable due to noise (Supplementary Information Figure SI-3), although the overall pattern remains the same as the fifty-trial window. We may not be able to pinpoint the exact switch point but with our current analysis and by testing multiple thresholds, we can generate a consistent and reliable measure for the model acquisition rate of each model and participant.

In addition to selecting the simplest, most-viable function, the simpler functions also enjoyed faster acquisition rates for different switch combinations (Figure 4). Therefore, simpler functions have an advantage both in the extent of selection and also in their rates of selection. Both these independent measures suggest that the learning of simpler models is preferred over that of equally viable complex models. The Occam's razor principle states that when multiple models are equally capable of explaining a dataset, the simplest one should be selected. Recent studies have revealed that humans exhibit Occam's razor-like parsimony in perceptual and sensorimotor learning tasks (Gershman, 2013; Genewein and Braun, 2014). If the Occam's razor is a pervasive principle in human perception and model learning, it may explain the previous findings on primacy of linear function over non-linear functions, since linear models would have low parametric complexity. Such a unifying principle could help us better understand how humans abstract and learn functions among variables in the world.

In summary, we propose that structure learning may serve as a unifying framework for function learning. We believe this to be a viable framework due to the results in Experiment 1 where function abstraction seems most likely. The separation of function learning and parameter learning makes room for abstraction, rule-based learning as well as associative accounts. Further, it helps us to understand human perceptual principles free of algorithm-induced biases and in a massively parallel manner. In this study, it has led to the revelation that human function learning obeys the Occam's razor principle.

### Conflict of interest statement

The authors declare that the research was conducted in the absence of any commercial or financial relationships that could be construed as a potential conflict of interest.
