# Supplementary material for: Structure learning and the Occam's razor principle: a new view of human function acquisition
Source: Front Comput Neurosci. 2014 Sep 30;8:121. doi: 10.3389/fncom.2014.00121 (PMC4179744; doi:10.3389/fncom.2014.00121)
Supplement: Supplementary file 1 [file DataSheet1.PDF]

7. Supplementary Material

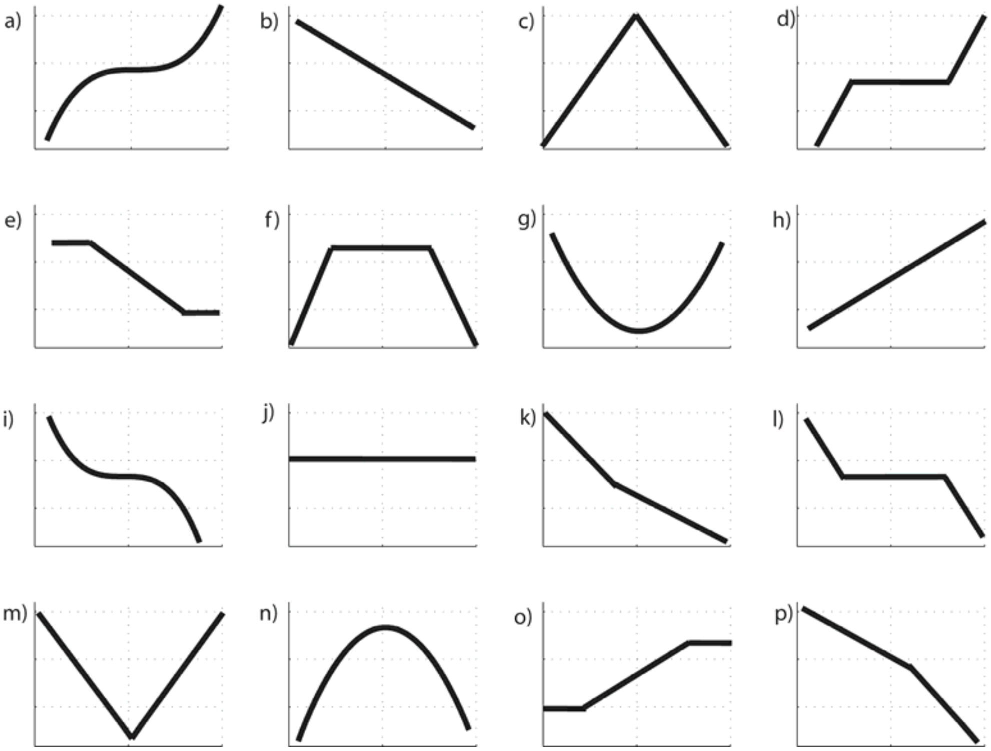

SI Figure 1: Questionnaire for experiment 1. Sketches of 16 possible perceived relationships between the cued target location and the target timing. Participants were asked to select the three that they experienced in correct order.

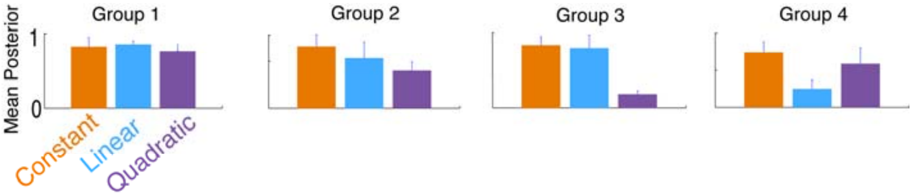

SI Figure 2: Inter- and within group variability: Within the duration that each model was presented, the posterior values for that model were averaged for each participant. The bars indicate the mean and standard error (non-gaussian) for how much this value varied for participants within each group. Unlike other analyses, these averages include the first 100 trials of the experiment where learning may have been incomplete. These values reflect the extent and not rate of model acquisition across participants.

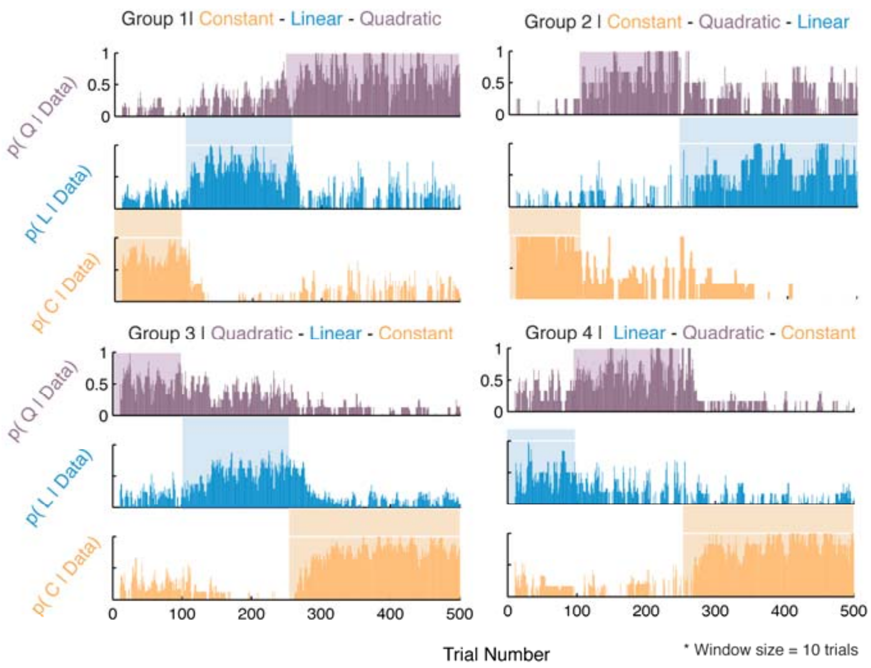

Supplementary figure 3: Function learning in Experiment 2 with a 10 trial window. Average posterior probabilities for the three functions given data within the windows. Color scheme and axes identical to Figure 2b. The four panels represent the four groups of participants.
